# Supplementary material for: Testing the Effectiveness of a Commercially Sold Probiotic on Restoring the Gut Microbiota of Honey Bees: a Field Study
Source: Probiotics Antimicrob Proteins. 2023 Dec 19;17(3):991–1000. doi: 10.1007/s12602-023-10203-1 (PMC12055933; doi:10.1007/s12602-023-10203-1)
Supplement: Supplementary file 3 — Supplementary file3 (PDF 5369 KB) [file 12602_2023_10203_MOESM3_ESM.pdf]

Supplementary material

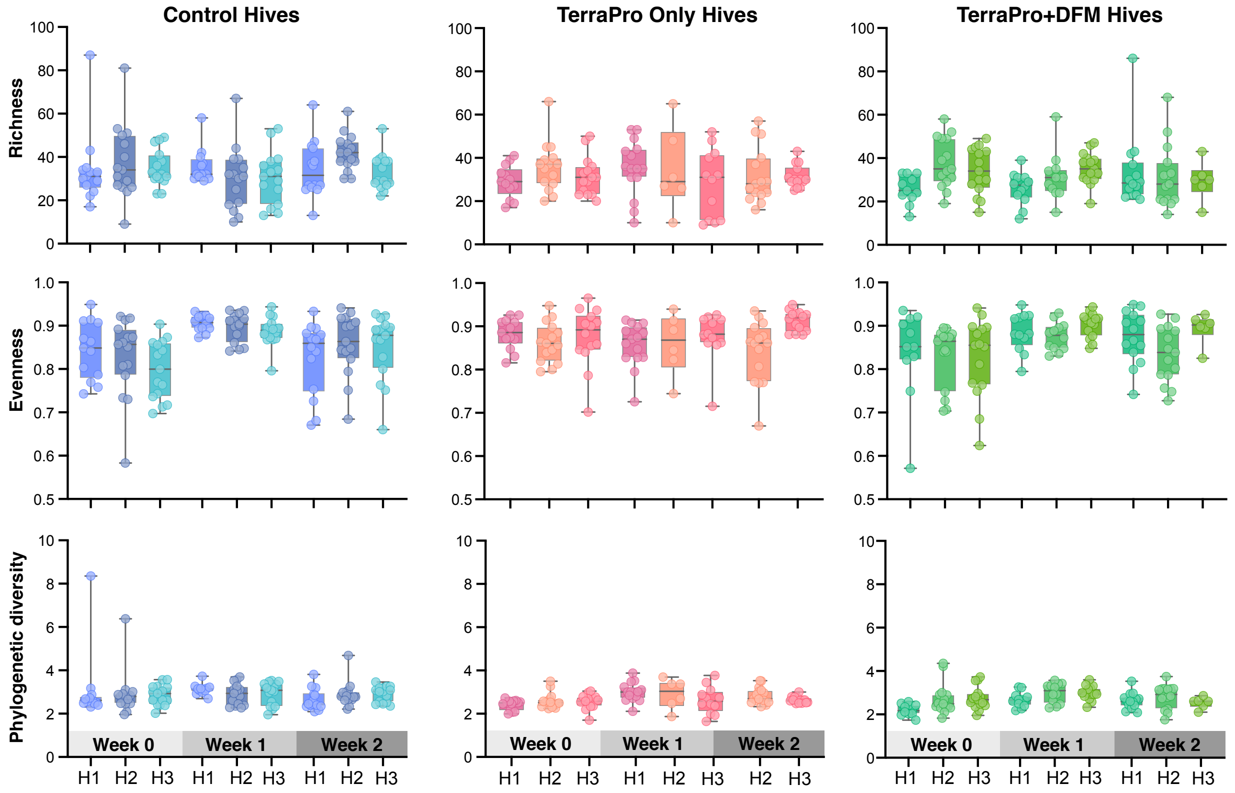

Figure S1. Alpha diversity comparisons of the gut microbiota of Control, Terra-Pro-Only, and Terra-Pro+DFM group hives at Week-0 post-Terra-Pro pre-DFM, Week-1 post-Terra-Pro post-DFM, and Week-2 post-Terra-Pro post-DFM. Alpha diversity metrics were based on richness (# of ASVs), evenness (Pielou's Evenness index), and phylogenetic diversity (Faith's Phylogenetic Diversity index). Alpha diversity significance (*P-value*) was determined using the Kruskall-Wallis test with Benjamini-Hochberg FDR correction (*Q-value*), see Dataset S1 for *P* and *Q* values.

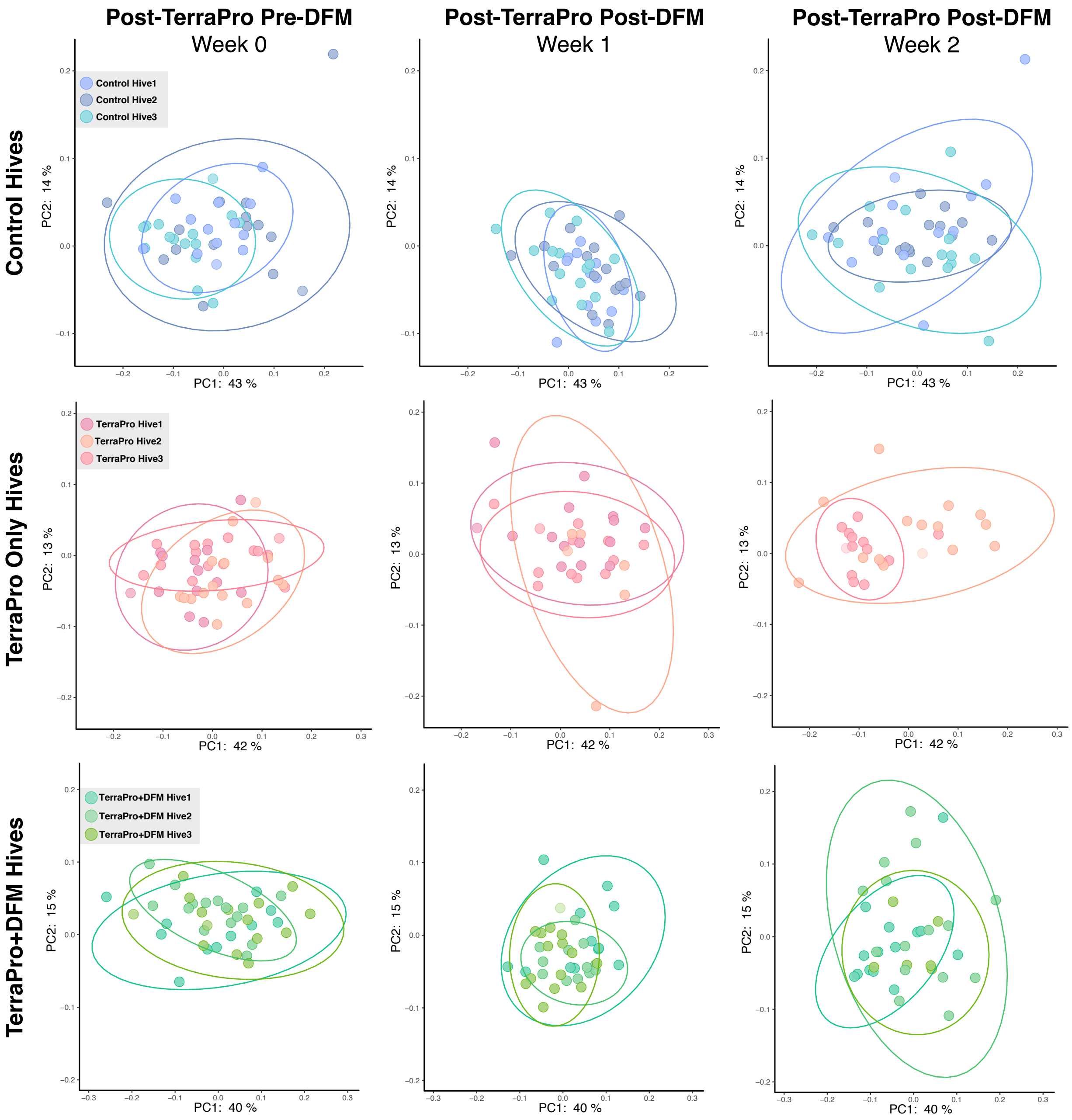

Figure S2. Beta diversity comparisons of the gut microbiota of control, Terra-Pro-Only, and Terra-Pro+DFM bees at Week-0 post-Terra-Pro pre-DFM, Week-1 post-Terra-Pro post-DFM, and Week-2 post-Terra-Pro post-DFM. Beta diversity was based on community similarity (weighted UniFrac) and visualized via principal coordinate analysis (PCoA) plots. Significance was tested using PERMANOVA with 999 permutations followed by Benjamini-Hochberg FDR correction (see Dataset S1 for *P* and *Q* values). Ellipses represent the 95% confidence interval.

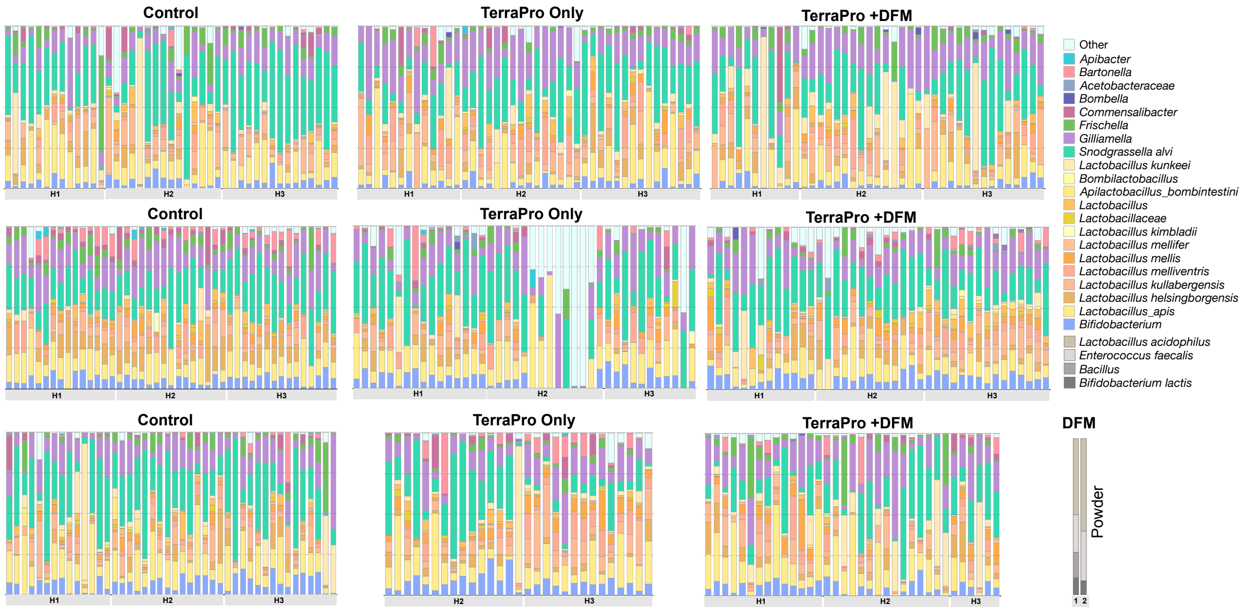

Figure S3. Bar plots representing the relative abundance of bacterial taxa found in individual bee samples from each experimental group, hive, and sampling timepoint. The DFM powder (probiotic) was also sequenced as a positive control.
